# Increased activation in the bilateral anterior insulae in response to others in pain in mothers compared to non-mothers

**DOI:** 10.1038/s41598-021-02162-w

**Published:** 2021-11-23

**Authors:** Irene Sophia Plank, Catherine Hindi Attar, Stefanie L. Kunas, Isabel Dziobek, Felix Bermpohl

**Affiliations:** 1 Department of Psychology, Institute of Life Sciences, Humboldt-Universität Zu Berlin, Berlin, Germany; 2 Berlin School of Mind and Brain, Humboldt-Universität Zu Berlin, Berlin, Germany; 3 Department of Psychiatry and Neurosciences | CCM, Charité – Universitätsmedizin Berlin, Corporate Member of Freie Universität Berlin and Humboldt-Universität Zu Berlin, Berlin, Germany; 4 Einstein Center for Neurosciences, Charité – Universitätsmedizin Berlin, Corporate Member of Freie Universität Berlin and Humboldt-Universität Zu Berlin, Berlin, Germany

**Keywords:** Neuroscience, Emotion, Social behaviour, Social neuroscience

## Abstract

Empathy allows us to share emotions and encourages us to help others. It is especially important in the context of parenting where children’s wellbeing is dependent on their parents’ understanding and fulfilment of their needs. To date, little is known about differences in empathy responses of parents and non-parents. Using stimuli depicting adults and children in pain, this study focuses on the interaction of motherhood and neural responses in areas associated with empathy. Mothers showed higher activation to both adults and children in pain in the bilateral anterior insulae, key regions of empathy for pain. Additionally, mothers more strongly activated the inferior frontal, superior temporal and the medial superior frontal gyrus. Differences between adult and child stimuli were only found in occipital areas in both mothers and non-mothers. Our results suggest a stronger neural response to others in pain in mothers than non-mothers regardless of whether the person is a child or an adult. This could indicate a possible influence of motherhood on overall neural responses to others in pain rather than motherhood specifically shaping child-related responses. Alternatively, stronger responses to others in pain could increase the likelihood for women to be in a relationship and subsequently to have a child.

## Introduction

Humans' emotional capacity for social understanding of others is vital to a social society and prosocial behaviour^1^. Social understanding of others is comprised of several processes^2^. One of these processes is empathy which according to de Vignemont and Singer is an isomorphic emotional response to other's emotions. Additionally, the person feeling empathy is aware of the other person being the source of the emotion^3^ (however, this is not without controversy, see for example^4^). Theories suggest that empathy is an important part of understanding others on an emotional level and part of the affective route of social understanding^2^. In this usage, it is differentiated from cognitive social understanding which refers to inferential processes to understand other's emotions and mental states. Competence in cognitive and affective social understanding is largely independent of one another^5,6^. According to Panksepp’s theory of emotions, ancient emotional circuits motivate and drive our behaviour. Social emotions, play, care and lust, are especially relevant for parental behaviour^7^. While Panksepp focussed on the role of opioids for attachment^8^, nowadays theories have shifted to oxytocin being an important base of not only attachment but parental behaviour^9–14^. Some evolutionary theories even propose that a general capacity for empathy developed out of parenting due to the increased duration of dependency in humans^10,15^. While there is a prolonged dependency in all mammals, this is especially pronounced in humans and not only includes care for survival but also education for adaptive social behaviour^10,16^. Parents who were empathetic towards their children increased their children’s likelihood to successfully procreate, thereby leading to the evolution of empathy. The mechanisms leading to empathy in parents in the first place could very well still be at play nowadays and lead to increased empathy responses especially to children in parents.

Several studies show a positive effect of empathic and compassionate parenting on one's offspring. Parental trait empathy is positively related to a child's attachment security^17^ and parents serve as important social models for children teaching them social competencies^18,19^. Parenting by nature forces people to use cognitive and affective social understanding constantly when interacting with their child which could lead to a training effect^20^. When asked to describe infants' behaviour in a video, parents are more likely to describe mental states compared to non-parents^21^. A recent study investigated the interaction of parenthood and trait empathy as measured by the interpersonal reactivity index questionnaire^22^. The authors found increased empathic concern in parents within 24 h of the birth of their child as compared to non-parents. In an attentional capture paradigm, infant faces led to slower response times than adult faces in women and this was more pronounced in mothers compared to non-mothers^23^. These findings were replicated and extended to pre-adolescent faces in distress^24^. In sum, these studies show that parents react differently to infants and children than non-parents.

Differences in social understanding due to parenthood also extend to the neural level. Proverbio and colleagues reported higher sensitivity for differences in facial affect of infants in parents as reflected in the N2^25^. Zhang and colleagues revealed increased activity in mothers in several frontal and occipital areas compared to non-mothers when viewing infant faces^26^. Interestingly, differences in emotional face processing between parents and non-parents may extend to adult stimuli^27^, although other studies only found differences concerning child faces^28^. Concerning affective stimulus material, Parsons et al. compared mothers’ and non-mothers’ brain responses to adults' or children's distressed or neutral sounds^29^. Pooling distressed and neutral sounds, they showed that infant stimuli led to stronger activation in multiple areas, including the left amygdala and orbitofrontal cortex. Adult stimuli, in turn, activated the left middle frontal gyrus more strongly than infant stimuli. Contrasting mothers with non-mothers revealed higher activation for mothers in several areas, including the right middle frontal gyrus, precuneus, middle temporal gyrus as well as left superior temporal pole and the orbitofrontal cortex. Taken together, research has shown that parenthood can have effects both on a behavioural and on a neural level even when unknown adults or children were the targets^23,26–29^. It is still unclear whether the influence of parenthood extends to empathy responses and in what way the empathy response in parents and non-parents is different when reacting to children as compared to adults.

Research has shown that observing others in pain and feeling pain yourself leads to considerable overlap in brain activation, for example in the bilateral anterior insulae (AI) and medial areas of the prefrontal cortex^30–32^. This overlap could indicate that we *feel with* the other person when we observe them in pain which is corroborated by correlations between activation in these areas and trait empathy or behavioural empathy ratings^30,33–36^. Empathy for pain paradigms investigate responses to other’s in pain and are open to influences of the perceiver^37^ and the person in pain^38,39^. These kinds of paradigms have two advantages over behavioural measures of empathy: first, it does not encourage participants to cognitively reflect on their empathy responses, thereby altering their natural, spontaneous response. Second, it is less strongly influenced by a tendency to feel and answer in a socially desirable manner through which a more objective measure of empathy is possible. This makes empathy for pain paradigms well-suited and well-established to investigate nuances in neural responses to others in pain like differences due to motherhood or comparing responses to children and adults in pain.

This study aimed to address the influence of motherhood of the perceiver and age of the protagonist (adults and children) of painful and neutral scenarios on neural responses as well as the interaction of both factors. The sample focussed on mothers because they provide primary care for children in most cases in Germany^40,41^. Additionally, although previous studies reported vast overlaps and similarities in fathers and mothers, they also indicated possible differences that justify separate investigations^42,43^. Specifically, this study aimed to answer the following questions: First, does motherhood interact with neural responses to painful over neutral scenarios in women? Second, are neural responses to painful over neutral responses larger if the protagonist is a child rather than another adult? Third, do mothers relative to non-mothers have stronger brain responses to children in pain? To answer these questions, we measured increased neural responses to children and adults in painful as opposed to neutral situations in mothers and non-mothers. In previous studies, this increased response has been associated with empathy^30,33–36^ We hypothesised (1) that mothers show stronger neural responses in areas associated with empathy to people in pain in general, (2) that children compared to adults in pain lead to higher neural responses in areas associated with empathy across groups and (3) that the increase of activation in areas associated with empathy in response to children is even more pronounced in mothers.

## Methods

### Participants

Women were recruited online and with flyers and had to fulfil the inclusion criteria of being healthy, right-handed, cisgender female, between 25 and 50 years old and having sufficient knowledge of German. Mothers had at least one biological child between 4 and 10 years of age while non-mothers were nulliparous and stated that they had no close and regular contact with children (neither professionally nor in their personal life). One non-mother reported an abortion and another an early miscarriage. Based on power analyses for a within-between interaction in a repeated-measures ANOVA with two groups and two measurements using G*Power (*f* = 0.25, *α* = 0.05, (1 − *β*) = 0.95, corr = 0.5)^44^, we aimed for a sample size of 54 women (27 mothers). Of the full datasets we obtained of women fulfilling the inclusion criteria, we excluded two participants due to artefacts that required neurological evaluation and two because they rated both painful and neutral stimuli as not painful. Due to the outbreak of Covid-19, we did not replace them. The final analysed sample consisted of 50 women (25 mothers, mean age mothers = 38.28; non-mothers = 35.64) and posthoc power calculations indicated a power of 0.93 for a within-between interaction in a repeated-measures ANOVA in G*Power ~ 3 (*f* = 0.25, *α* = 0.05, *n* = 50, corr = 0.5). Mothers and non-mothers did not differ in age, intelligence or socio-economic status (see Table 1). The study was approved by the Ethics committee of the Charité – Universitätsmedizin Berlin and was conducted following the Code of Ethics of the World Medical Association (Declaration of Helsinki). All women received monetary compensation and gave written informed consent before participating.

**Table 1:** Mean, standard error and corrected Bayes factor for all Bayesian Mann–Whitney-U tests performed on answers from the interview and scores from the questionnaires. The proportion of single women per group and Bayes factor of the contingency table.

Measurement	Mothers	Non-mothers	*BF*_*10*_
Age	38.3 (± 0.8)	35.6 (± 1.4)	0.179
Importance of having children (0–4)	3.7 (± 0.1)	2.7 (± 0.3)	0.443
MinIQ	27.7 (± 2.1)	30.9 (± 2.2)	0.107
Mood state (0–4)	3.1 (± 0.1)	3.1 (± 0.1)	0.810
ECR-RS	23.9 (± 1.4)	31.4 (± 1.8)	1.993+
ERQ	41.0 (± 1.3)	42.4 (± 1.4)	0.091
IRI-emp	44.9 (± 1.1)	43.1 (± 1.4)	0.119
IRI-PT	15.3 (± 0.4)	15.1 (± 0.4)	0.083
KSE-G	1.8 (± 0.1)	2.0 (± 0.1)	0.403
Relationship status: single (proportion of group)	20%	76%	975***
SES	14.5 (± 0.6)	14.0 (± 0.7)	0.091
TAS	39.0 (± 1.8)	40.2 (± 1.9)	0.081

*ECR-RS* relationship attachment, *ERQ* emotion regulation quotient, *IRI* interpersonal reactivity, *emp* empathy, *PT* perspective taking, *KSE-G* tendency for social desirability, *SES* socioeconomic score, *TAS* alexithymia score; + signifies anecdotal evidence, ***signifies decisive evidence (according to Jeffrey's scheme ^56^).

### Experimental procedure

Prior to scanning, participants read the study information with the consent form and had the opportunity to ask questions. Then, one of the experimenters conducted a semi-structured interview with the participants about their demographics, health, mood state, family and relationship to children. Within this interview, participants were asked about their mental health history and participants who reported repeated or mental health problems within the last two years were excluded. After the interview, participants performed a short IQ screening^45^ followed by questionnaires to measure emotion regulation (ERQ^46^), relationship attachment (ECR-RS^47^), social desirability (KSE-G^48^), alexithymia (TAS-20^49^) and trait empathy (IRI^50^). The empathy for pain fMRI task was presented in two runs of 8.5 min. Additionally, participants performed another task in the scanner as well as a run of resting-state fMRI. Scanning lasted about 60 min, and the total experiment duration was about 90 min.

### Experimental paradigm

**Figure 1:**
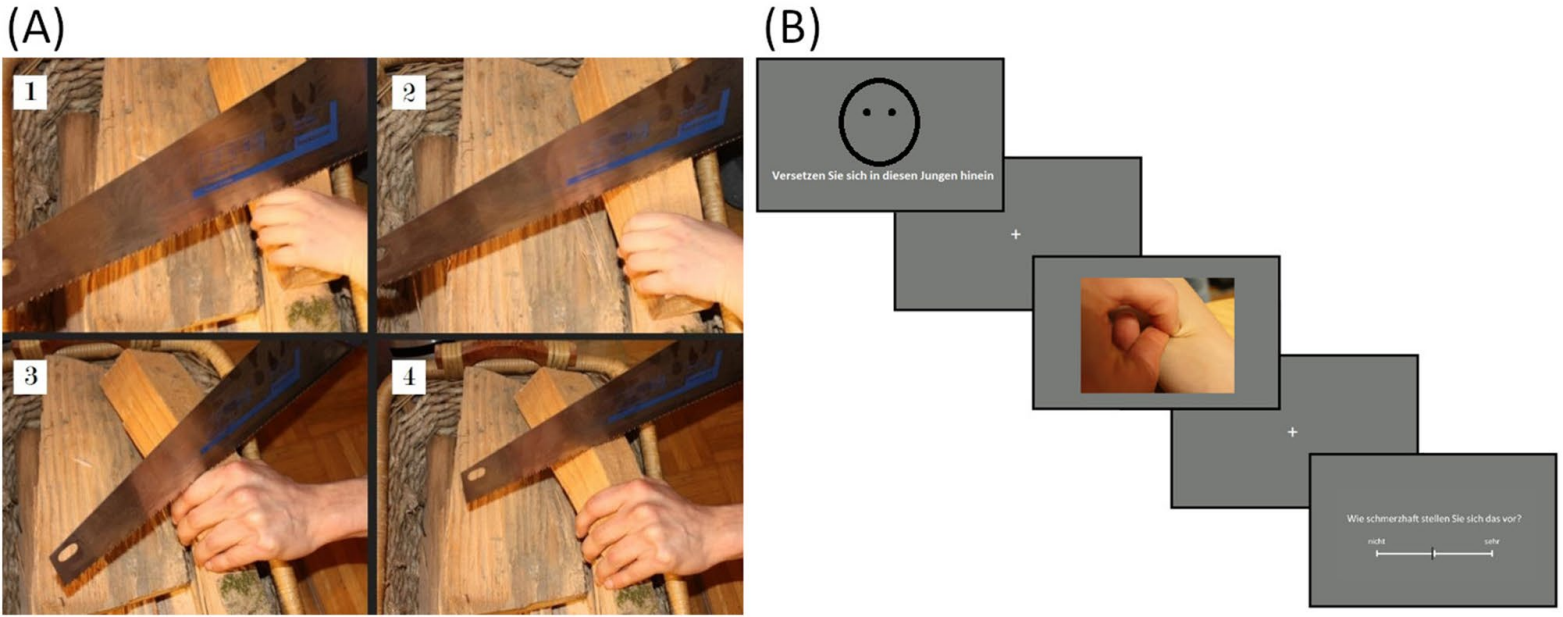
(**A**) Example for a scenario in all four variations: 1. child painful, 2. child neutral, 3. adult painful, 4. adult neutral. (**B**) Schematic of the neutral introductory face including a term for adult or child and the first picture of a trial followed by a rating.

For this study, we adapted a paradigm used by Lamm and colleagues to compare two groups of people in painful versus neutral scenarios^51^, children and adults respectively. Participants viewed colour pictures of situations shown in four variations: painful for a child, neutral for a child, painful for an adult and neutral for an adult (see Fig. 1A). Neutral scenarios also contained the cause for the pain but without showing the actual painful situation. In total, 31 situations were created and photographed. The pictures were assessed by 22 subjects for imagined painfulness and matching of the scenarios. We chose 24 situations where the scenarios were rated as well-matched and had a difference between painful and neutral scenarios of at least 30 on a scale from 0 to 100. This resulted in 24 stimuli per condition and 96 stimuli in total. There was no difference in rating between the child and adult stimuli. All pictures were matched in colour, luminance and contrast. Four neutral faces were chosen to introduce the scenarios and emphasised the protagonist of the following scenarios since differences between scenarios featuring children and adults were deliberately subtle to ensure appropriate matching (female and male adult from the KDEF set^52^, 4-year-old female and 5-year old male child from the CAFE set^53,54^).

Each trial started with a face presented for 3 s followed by four pictures of scenarios, two painful and two neutral, presented for 3.5 s each (see Fig. 1B). The order of these four pictures was randomised. The participants were asked to imagine that the person whose face they saw was the protagonist of the displayed situations and to put themselves in their shoes. They were told that in the case of a painful scenario, the protagonist would be receiving the pain. Pictures of scenarios were preceded by a white fixation cross (duration from truncated exponential, *λ* = 1.01, mean = 2.5 s, min = 2.0 s). After 50% of the pictures, they were asked to rate how painful they imagine this scenario to be on a continuous rating scale ranging from “not” to “very” with two buttons using their index and ring finger of the right hand. Importantly, these ratings are not empathy ratings as they ask to rate the imagined painfulness and not the resulting feeling. Their purpose is to assess whether possible differences in responses are due to differences in interpretation of the painfulness of the scenarios. Participants had 3 s to rate the imagined painfulness without having to confirm their answer. Each rating was separated from the corresponding picture with a fixation cross for an average of 1.3 s. Between trials, empty grey screens were presented for 4–7 s (average 4.8 s). Pictures were presented in a pseudo-randomised order with the restriction that corresponding painful and neutral scenarios were not presented close to each other. Additionally, there were never more than two trials of the same protagonist presented after each other.

### Sample characteristics and imagined painfulness

All analyses here were performed in JASP (scripts and data: https://osf.io/kyr8v/)^55^. Evidence was labelled as anecdotal, moderate, strong, very strong or decisive based on the adaptation of Jeffrey's scheme used in JASP^56^. Bayesian Mann–Whitney-U tests were computed with 10,000 random samples to compare mothers and non-mothers in age, socio-economic status (SES^57^), intelligence score^45^, the importance of having children, questionnaire scores and mood state. All Mann–Whitney-U tests were corrected for multiple comparisons using Westfall's method^58,59^. Bayesian contingency tables were used to compare relationship status between mothers and non-mothers. Imagined painfulness during the fMRI paradigm was analysed with a 2 × 2 × 2 Bayesian mixed ANOVA with factors motherhood, painfulness and protagonist (child or adult). Variables that showed at least moderate evidence for a group difference in the Bayesian Mann–Whitney-U tests would have been added as covariates to the null model, however, none fulfilled the criterion. We calculated partial eta-squared to measure the effect size of the predictors and the interactions of the ANOVA by sampling 500 times from the posterior predictive distribution and calculating a median and a confidence interval for each predictor and the interactions.

### Neuroimaging data acquisition

All scans were acquired using a 20-channel 3 Tesla MRI (Siemens Magnetom Prisma, Siemens Medical Solutions, Erlangen, Germany) at the Berlin Center for Advanced Neuroimaging. Functional images were acquired as T2*-weighted echo-planar images (EPI) across six runs, two of which have been analysed for this paper. Each of the analysed runs consisted of 256 scans acquired in 32 consecutive slices of 3 mm (voxel size = 3 mm^3^, TR = 2000 ms, TE = 30 ms, FA = 78°, FOV = 192 mm). Before the functional runs, a structural image was acquired using a T1-weighted magnetically prepared rapid acquisition gradient echo with a 1 mm^3^ voxel size (176 slices, TR = 2539 ms, TE = 4.94 ms, FA = 7°, FOV = 256 mm). Between the structural and the functional images, field maps were acquired for each participant (32 slices á 3 mm, TR = 400 ms, TE1 = 5.19 ms, TE2 = 7.65 ms, FA = 60°, FOV = 192 mm).

### Neuroimaging data preprocessing

All images were preprocessed using *fMRIPrep* 20.0.6^60,61^ which is an automated preprocessing pipeline (for an extensive description of all steps applied by *fMRIPrep* see the supplementary materials). Anatomical images were corrected for intensity non-uniformity and used as T1 weighted references which were skull-stripped and segmented into tissue types. After surface reconstruction, brain masks were refined and spatially normalised to the Montreal Neurological Institute space (MNI152NLin2009cAsym^62^). For each run of functional images, susceptibility distortion based on fieldmaps, coregistration, realignment, slice time correction and normalisation were performed. Participants who moved more than one voxel (3 mm) were excluded from the analysis. The preprocessed images from *fMRIPrep* were subsequently detrended based on a linear model of global signal^63^ to remove global effects from the time series. This method is efficient in improving the signal-to-noise ratio^58^, but see also^59^. Then, images were continued to be smoothed in SPM12 (Wellcome Department of Imaging Neuroscience, University College London, UK, 2014) using a full width half maximum of 6 mm of the Gaussian smoothing kernel. All images were masked with participants' brain masks.

### Neuroimaging analysis

All univariate analyses of the fMRI data were performed using the general linear model as implemented in the SPM12 software. For each subject, one first-level GLM including both runs was specified for the total duration of the pictures in the four conditions shown in Fig. 1A. A “painful > neutral” contrast was computed separately for adult and child stimuli (scripts and contrasts: https://osf.io/kyr8v/). Group-level differences were assessed using a flexible factorial design specification based on the contrast images computed in the first-level analysis. The factors of interest were motherhood (between) and protagonist (within) as well as their interaction. To test the hypotheses, a region-of-interest (ROI) analysis was performed using a single mask for small volume correction. We chose our ROIs based on a recent meta-analysis to focus on specific parts of areas functionally associated with empathy for pain^64^. ROIs are spheres of 10 mm around centres in the medial superior frontal gyrus (mSFG: − 2, 24, 38; other studies have reported the adjacent anterior cingulate cortex instead^30,65^), left supramarginal gyrus (lSMG: − 62, − 22, 32) and bilateral AI (left AI: − 38, 16, − 4; right AI: 44, 8, − 4) to create one mask containing all four spheres^64,66^. A whole-brain analysis was performed with a cluster-level threshold of family-wise error (FWE) corrected *p* < 0.05 to explore additional differences. All contrasts were masked with a 10% probability grey matter mask provided by SPM12. We also computed a multiple regression to explore a possible relationship between the imagined painfulness ratings and the neural response to painful over neutral scenarios. Furthermore, we explored possible structural differences using voxel-based morphometry (VBM) as implemented by CAT12 running on SPM12^67^. We used a two-sample t-test including the covariates age and total cranial volume to compare mothers and non-mothers.

## Results

### Sample characteristics

Comparisons of questionnaire scores and answers in the interview showed that mothers and non-mothers were comparable in most aspects (see Table 1). However, mothers were more likely to be in a relationship than non-mothers with a Bayesian Contingency Table showing decisive evidence in favour of the alternative hypothesis. While only 20% of mothers were single, 76% of the non-mothers were single (*BF*_*10*_ = 975). Additionally, there was anecdotal evidence that mothers gave higher relationship attachment ratings than non-mothers as measured with the ECR-RS (*BF*_*10*_ = 1.993).

### Imagined painfulness

**Figure 2:**
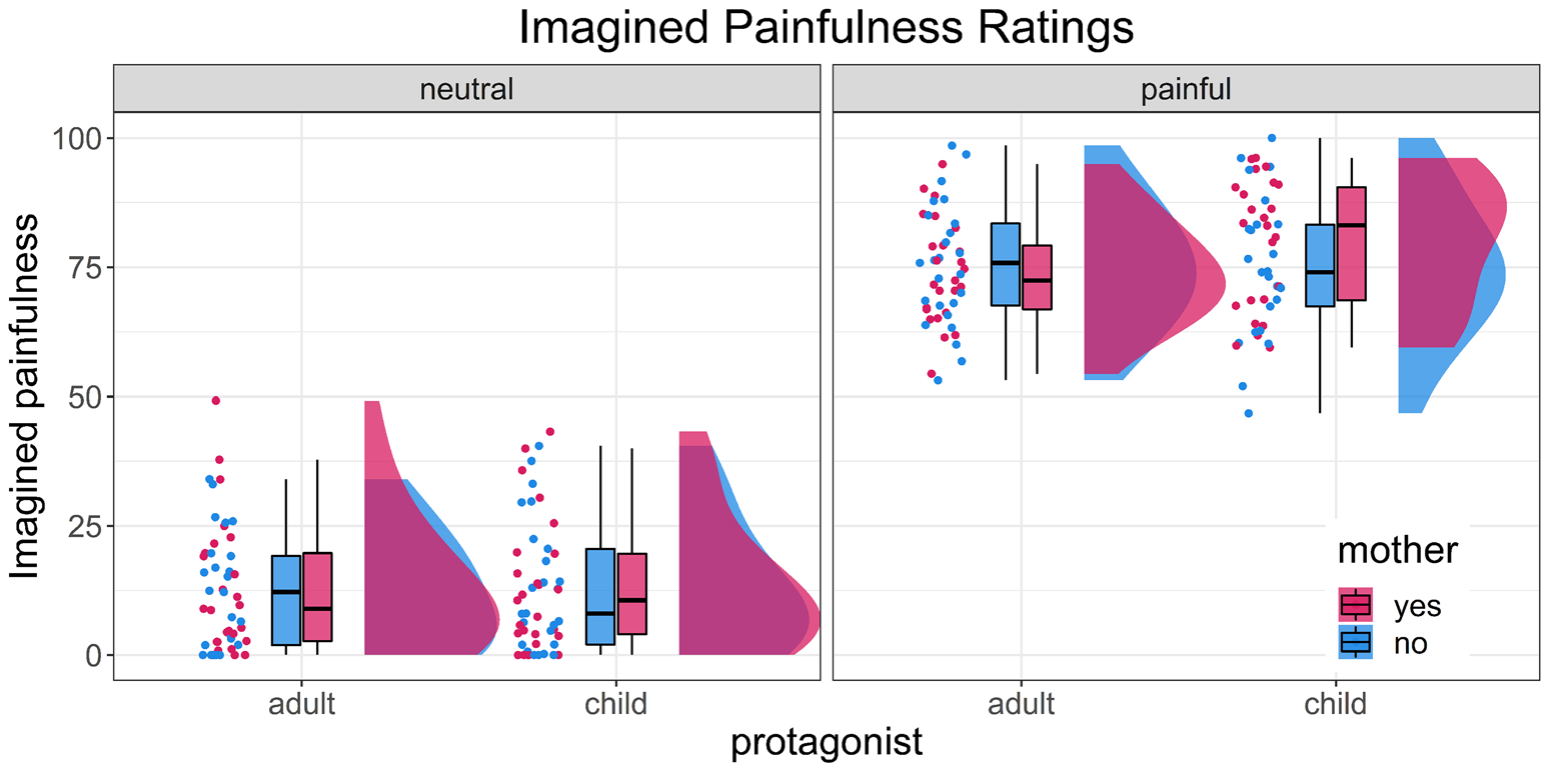
Imagined painfulness rated during the fMRI paradigm. The imagined painfulness of half of all stimuli was rated on a continuous scale from 0 to 100.

The Bayesian mixed ANOVA determined that the model which represents the data best only includes the main factor of imagined painfulness. This model shows decisive evidence in favour of the alternative hypothesis that pictures that were used as painful stimuli were indeed rated higher on the imagined painfulness scale than neutral scenarios (*BF*_*10*_ = 3.3 * 10^91^). The median effect size *η*_*p*_^*2*^ of the predictor pain was 0.87 which constitutes a large effect (*CI*_*90%*_ = 0.85–0.90)^68^. Adding other factors or interactions did not improve the model and the confidence intervals of the effect size included zero (see Table S1 in the supplementary materials). Inclusive Bayes factors for each factor separately over all models show decisive evidence *for* the inclusion of pain but moderate evidence *against* the inclusion of protagonist or mother. Therefore, the experimental manipulation was successful but there were no differences between mothers and non-mothers in how painful a stimulus was imagined and no difference due to the protagonist of the stimulus (i.e., adult or child) or the interaction of both factors (see Fig. 2).

### Neuroimaging results

Both mothers and non-mothers reliably exhibited increased activation in areas associated with empathy in response to the painful as compared to the neutral scenarios (see Table S2and Figure S1in the supplementary materials). The region-of-interest (ROI) analysis confirmed stronger neural responses to painful over neutral scenarios for mothers relative to non-mothers in the bilateral AI (left: *t* = 5.59, *k*_*E*_ = 124, *CI*_*90%*_ of *CE* = 0.37–0.68; right: *t* = 4.43, *k*_*E*_ = 81, *CI*_*90%*_ of *CE* = 0.54–1.19). There were no activation differences between mothers and non-mothers in the mSFG or lSMG. Adult and child stimuli did not lead to differences in activations in any of the ROIs. There was also no indication for the interaction of both factors in the ROI analysis. We additionally conducted the ROI analysis excluding two non-mothers due to a previous pregnancy. The results mirror the results of the whole sample with increased activation in mothers compared to non-mothers in the bilateral insulae and no significant differences in any other contrast (left: *t* = 5.37, *k*_*E*_ = 104, *CI*_*90%*_ of *CE* = 0.36–0.69; right: *t* = 4.48, *k*_*E*_ = 79, *CI*_*90%*_ of *CE* = 0.57–1.23).

**Figure 3:**
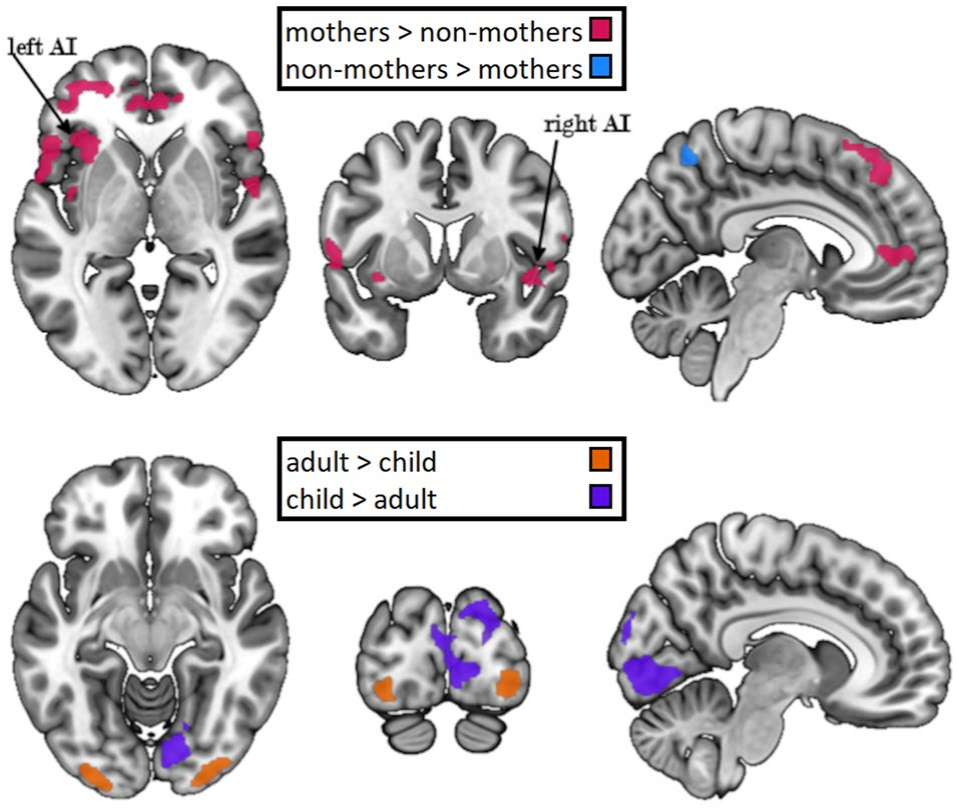
Results of the four main contrasts in the whole-brain analysis with a cluster corrected threshold of *pFWE* < 0.05. Images have been created with MRIcroGL ^70^.

The whole-brain analyses showed additional differences in activation patterns between mothers and non-mothers as well as in responses to child and adult stimuli, however, no interaction between the two (see Table 2 and Fig. 3). Mothers had a higher response to painful over neutral scenarios than non-mothers in frontal areas, including the bilateral inferior frontal gyrus (IFG) extending into the bilateral insulae and right medial SFG, as well as the right superior temporal gyrus (STG) and cuneus, left cerebellum and rolandic operculum (RO). Non-mothers relative to mothers showed a stronger neural response to painful over neutral scenarios in the left superior parietal gyrus (SPG).

**Table 2:** Results of the whole-brain analysis based on the flexible factorial model. All contrasts are t-contrasts based on the flexible factorial model. All contrasts are t-contrasts based on differential first-level contrast images of “painful > neutral” scenarios.

	BA	H	*k*_*E*_	*t*-value	*x*	*y*	*z*	*CI*_*90%*_* of CE*
**Mothers > non-mothers**								
Inferior frontal gyrus inc. anterior insula	45	L	720	6.80	− 51	22	− 1	0.43–0.71
	44			6.14	− 57	8	2	
	47			5.75	− 35	26	− 5	
Superior temporal gyrus inc. insula	22	R	1008	6.31	46	− 33	14	0.31–0.53
	4			6.20	42	− 5	8	
	40			5.72	52	− 17	12	
Middle frontal gyrus	10	L	380	6.26	− 41	52	− 5	0.46–0.78
	10			5.18	− 23	52	− 3	
	10			4.71	− 37	50	10	
Cuneus	18	R	187	6.24	22	− 87	12	0.15–0.26
	18			4.96	18	− 99	10	
	18			4.29	16	− 93	20	
Inferior frontal gyrus	45	R	181	5.82	52	24	14	0.10–0.30
	45			5.31	54	26	− 3	
	47			4.56	48	24	− 9	
Inferior frontal gyrus	45	L	126	5.60	− 55	24	18	0.27–0.50
	45			4.85	− 47	24	16	
Superior frontal gyrus	8	M	124	5.55	− 5	38	48	0.23–0.42
	8			5.54	− 5	34	58	
	6			4.07	− 5	22	60	
Cerebellum (Crus-1)		L	124	5.26	− 23	− 79	− 27	0.27–0.52
				4.53	− 35	− 75	− 25	
Rolandic operculum	4	L	210	5.04	− 43	− 7	8	0.38–0.76
	1			4.74	− 39	− 17	16	
	13			4.34	− 35	− 9	14	
Orbital superior frontal gyrus	10	R	242	4.64	16	48	− 5	0.16–0.33
	10			4.61	4	46	2	
	32			4.50	− 7	46	2	
Superior frontal gyrus	9	R	224	4.47	14	54	28	0.22–0.48
	9			4.43	22	50	28	
	10			4.22	16	62	22	
**Non-mothers > mothers**								
Superior parietal gyrus	7	L	320	5.86	− 23	− 63	62	0.28–0.51
	7			5.60	− 9	− 63	58	
	7			5.45	− 37	− 63	58	
**Adults > children**								
Inferior occipital gyrus	18	R	239	5.97	36	− 87	− 9	0.70–1.24
Middle occipital gyrus	18	L	148	5.54	− 27	− 99	− 7	0.97–1.78
**Children > adults**								
Superior occipital gyrus	19	R	1020	6.13	22	− 91	30	0.67–1.16
	18			5.95	12	− 81	− 11	
	17			5.02	8	− 91	− 1	
Cuneus	18	L	123	4.89	− 11	− 77	30	0.74–1.48
	7			4.08	− 17	− 67	32	
Interaction	No clusters reached significance							

Cluster-corrected *p*_*FWE*_ < 0.05; all results are grey matter masked; *L* left, *R* right, *M* medial, *k*_*E*_ cluster size, *CE* cluster estimate (90% confidence interval), coordinates are in MNI space and are the location of the peak voxel for each cluster.

Both non-mothers and mothers had a stronger painful over neutral response to child compared to adult protagonists in the right superior occipital gyrus (SOG) and the left cuneus. For the opposite contrast, both groups showed higher activation in response to adult stimuli in the right inferior occipital gyrus and the left middle occipital gyrus.

There were no areas where the painful over neutral response was significantly correlated with the imagined painfulness ratings. The two-sample t-test of the VBM analysis revealed no structural differences between mothers and non-mothers indicating similar brain structures.

## Discussion

This study shows increased neural activation in mothers compared to non-mothers as a response to painful over neutral scenarios within the bilateral anterior insulae which are considered core regions for empathy for pain^30,31,64,65,70^. While previous studies have already shown increased emotional responses to infants' and prepubescent faces^24^ and infants' vocalisations^29^, this study extends current knowledge on differences between mothers and non-mothers to neural responses to others in pain in complex situations. Increased responses were found in mothers compared to non-mothers in the bilateral anterior insulae as core regions of empathy for pain^30,64^. This increased response in mothers was found regardless of mothers and non-mothers rating the imagined painfulness of the scenarios similarly indicating differences in their neural response in areas associated with empathy despite similar pain perception. Importantly, mothers reacted more strongly on a neural level not only to children but also adults in pain. There was no interaction between the motherhood of the perceiver and the person getting hurt in the scenario. Therefore, mothers showed stronger neural activation in areas associated with empathy in response to both children and adults in pain. Further, we compared grey matter volumes in mothers and non-mothers throughout the brain without finding any significant structural differences. These structural findings indicate that the functional group differences observed in the present study are not related to structural differences.

Importantly, enhanced neural activation of the AI has also been demonstrated for people in pain or suffering themselves^32^. This indicates a shared network for feeling with someone and feeling in general. AI activation has been found across different modalities suggesting that the AI is encoding the emotional effect and not the sensory features^71^. However, we have chosen not to include a behavioural measurement for empathy so that our participants are not cognitively reflecting on their empathy responses or pressured into behaving in a socially desirable manner, especially since this may have influenced our two groups to varying degrees. A vast body of literature links differences in activation in the anterior insulae between perceiving painful and neutral scenarios to experiencing empathy [e.g.,^30–33,37,38^]. It is, therefore, possible that the here observed differences in activation in areas associated with empathy may translate to higher empathy. However, alternative interpretations of the present findings are conceivable, e.g., differences in pain sensitivity, pain expectation, error prediction or other affective responses like revulsion, disgust or even arousal. These alternative interpretations were not regarded in the present and many former studies^35,51,72,73^. Therefore, future studies should investigate possible other influences besides empathy on the neural responses within the empathy for pain paradigm.

Ratings of imagined painfulness did not correlate with the neural response of each participant to painful over neutral scenarios. This indicates that no brain regions were associated with a physical assessment of painfulness. The neural response to painful over neutral scenarios that we compared between mothers and non-mothers was therefore independent of the physical assessment of painfulness. We also observed no differences in imagined painfulness between mothers and non-mothers, despite differences in their neural response to painful over neutral scenarios. This indicates that even though mothers and non-mothers assessed the physical pain the same, their reaction was different. If mothers and non-mothers had rated the imagined painfulness of the stimuli differently, their difference in interpretation of the imagined painfulness might have been the driving force. However, since there were no significant differences between the groups in the imagined painfulness of the stimuli, this indicates that faced with the same scenarios, mothers and non-mothers interpret the situation similarly but then react differently to it.

Contrary to our hypotheses, there were no differences between mothers and non-mothers in two of our regions of interest, namely the medial superior frontal gyrus and the left supramarginal gyrus. Bzdock and colleagues proposed that the medial superior frontal gyrus is implicated in the interpretation of complex social situations involving both affective and cognitive social understanding as well as moral cognition^74^. The left supramarginal gyrus is associated with higher-order somatosensory processing but is also active when inferring another person’s emotional state^75,76^. This could indicate that certain subprocesses of responses to painful over neutral scenarios that are subserved by these areas do not differ between mothers and non-mothers while other aspects subserved by the bilateral anterior insulae show differences. To investigate this possibility, paradigms need to be developed that allow manipulating subprocesses of responses to painful over neutral scenarios.

Additionally to differences in neural responses to painful over neutral scenarios in some regions associated with empathy, mothers also showed increased activation in other regions associated with social understanding compared to non-mothers. This included regions that a recent hierarchical analysis of social understanding associated with affective processes, like the triangular part of the IFG and the right STG, both extending into the insula. These areas have been linked to shared networks that are activated both when observing for example an emotional facial expression and producing it oneself^77^. This shared activation is assumed to facilitate the understanding of others^78,79^.

Furthermore, mothers showed a response to painful over neutral scenarios in regions commonly associated with predominantly cognitive processes of social understanding like the SFG, RO^77^ and the cerebellum^80^. Cognitive social understanding might have been more strongly involved due to the complexity and naturalism of the stimuli. The close matching between painful and neutral scenarios has the benefit that differences are not due to reactions to threatening stimuli. However, it also meant that participants had to closely pay attention to interpret the given scenario correctly. For example, some pictures contained needles. While in the neutral version, the needle was wearing a cap, it was still held close to the skin. Participants had to see the cap and infer that due to the needle being capped this contact between skin and needle is *not* painful. The differences in activation in these areas between mothers and non-mothers might indicate that mothers may have employed additional cognitive resources to interpret the stimuli and possibly understand the scenarios and the people in them better.

This study cannot determine the causal relationship between motherhood and neural responses to painful over neutral scenarios. On the one hand, the differences observed in this study could be due to the experiences of motherhood itself: caring for another human being who is not yet fully capable of expressing themselves and still dependent on their parent may mean that mothers increasingly rely on their empathy system. Therefore, the connections could be strengthened, leading to them exhibiting a stronger response to the same scenarios. Several studies have shown that social understanding can be increased by training^20,81^. Motherhood could have similar training effects simply due to everyday life situations with their children. On the other hand, it is possible that women with a stronger response to others in pain are more likely to be in a relationship, as observed in our sample, and become mothers. In this case, motherhood could have no effect on neural responses to others in pain. Since we do not have responses to others in pain prior to motherhood as a baseline, it is even possible that motherhood diminishes responses to others in pain for people other than their own children but responses are nonetheless larger than in non-mothers due to a higher baseline prior to motherhood^82^. Additional research is needed to disentangle these options from one another.

Surprisingly, activation in brain regions of interest was not modulated by the protagonist of the stimulus in women. All differences between adults and child stimuli were confined to the occipital lobe in areas most commonly associated with visual processing. Additionally, mothers also did not react more strongly to children than non-mothers did. Both missing effects might be due to the stimulus material: to keep adult and child stimuli comparable, only body parts and not faces were shown in the scenarios and scenarios were closely matched. As a consequence, the difference between child and adult scenarios were in some cases very subtle. We used faces to introduce the scenarios to explicitly point out the protagonist of the following scenarios, but the differences still might have been too subtle in our paradigm. Previous studies have shown that the empathy for pain paradigm employed here is sensitive to at least some differences due to the protagonist, for example, race^39^. However, most studies have used less complex stimuli and therefore participants might have focussed more on the protagonist while in this paradigm they could have focussed on the overall situation. Moreover, in most studies, out-group protagonists led to a diminished or reduced neural response^39^. In our study, adult protagonists were the in-group, but we expected increased responses to child stimuli. Children's special status may lead to them being treated as in-group instead of out-group but not to an additional increase in response. While neural responses to others in pain in areas associated with empathy did not differ between protagonists, differences might still surface when focussing on the consequences of these responses: children in pain could lead to more compassion and a stronger motivation to help. Our empathy for pain paradigm was designed to elicit a response by asking participants to imagine themselves in the scenarios. Therefore, compassion was not encouraged in this paradigm. Last but not least, higher neural responses to children in pain could be strictly confined to their own offspring^83^. Therefore, several possibilities could explain why activation in areas associated with empathy was not influenced by the protagonist in our paradigm. Further research is needed to investigate and disentangle these possibilities.

The mechanisms of the effect of motherhood should be subject of further scientific investigation. This study compared biological mothers to non-mothers who do not have close and/or frequent contact with children. This implied three factors that might operate independently of each other: biological parenthood, motherhood and contact with children. First, studies have found many similarities between adoptive and biological mothers, both in attention allocation^84^ and emotional reaction functions^85^. A study comparing biological and adoptive mothers who adopted their children as infants might find effects of motherhood on neural responses to others in pain similar to those found in the present study. Second, recent studies have started to investigate the influence that fatherhood has on men and how this compares to the influence of motherhood on women^42^. Therefore, it would be interesting to see if our results can be replicated in a sample of primary-care fathers. Third, some people made childcare their profession. Investigating kindergarteners, nannies and similar professionals who are not parents could answer the following questions: is it the responsibility for and care of children that leads to an increased neural response to others in pain? Or is it the special bond between parents and their children that fosters these differences? More research is needed to disentangle these factors and shed further light on the connection between parenthood, childcare and empathy. Some of these factors are connected with hormonal changes^11,86–90^, others could also be explained by training effects^20,91^. Apart from disentangling the underlying factors, studies going forward should examine the influence of these possible mechanisms.

This study indicates the possibility of differences in pain-related empathy between mothers and non-mothers. Using an empathy for pain paradigm, women were confronted with matched painful and neutral scenarios depicting children or adults as protagonists. Mothers exhibited a neural response to painful over neutral scenarios than non-mothers in core regions of empathy as well as areas associated with cognitive social understanding despite both groups giving the same ratings for imagined painfulness of the scenarios. Surprisingly, whether the protagonist of the stimulus was a child or an adult did not alter the neural response to painful over neutral scenarios outside of visual processing areas. There was also no interaction between the protagonist of the stimuli and motherhood. Therefore, mothers in this study showed a higher neural response to others in pain in areas associated with empathy than non-mothers regardless of the protagonist of the scenario. Although the directionality of this effect is still unclear, this difference in neural response to others in pain may be the basis for differences in various aspects of social understanding including compassion and motivation to help. Our results could inform courses and materials used to prepare women who are expecting for motherhood. Additionally, they demonstrate that parental status is an important factor to consider in research on social understanding and neural responses to others in pain.

## Supplementary Information

Supplementary Information.

## Data Availability

Data and scripts to reproduce the results are available in the Open Science Framework repository: https://osf.io/kyr8v/.
