# Supplementary material for: Increased activation in the bilateral anterior insulae in response to others in pain in mothers compared to non-mothers
Source: Sci Rep. 2021 Nov 23;11:22757. doi: 10.1038/s41598-021-02162-w (PMC8610985; doi:10.1038/s41598-021-02162-w)
Supplement: Supplementary file 1 — Supplementary Information. [file 41598_2021_2162_MOESM1_ESM.pdf]

Supplementary materials

Increased activation in the bilateral anterior insulae in response to others in pain in mothers compared to non-mothers

Irene Sophia Plank, Catherine Hindi Attar, Stefanie Kunas, Isabel Dziobek, Felix Bermpohl

Supplementary tables and figures

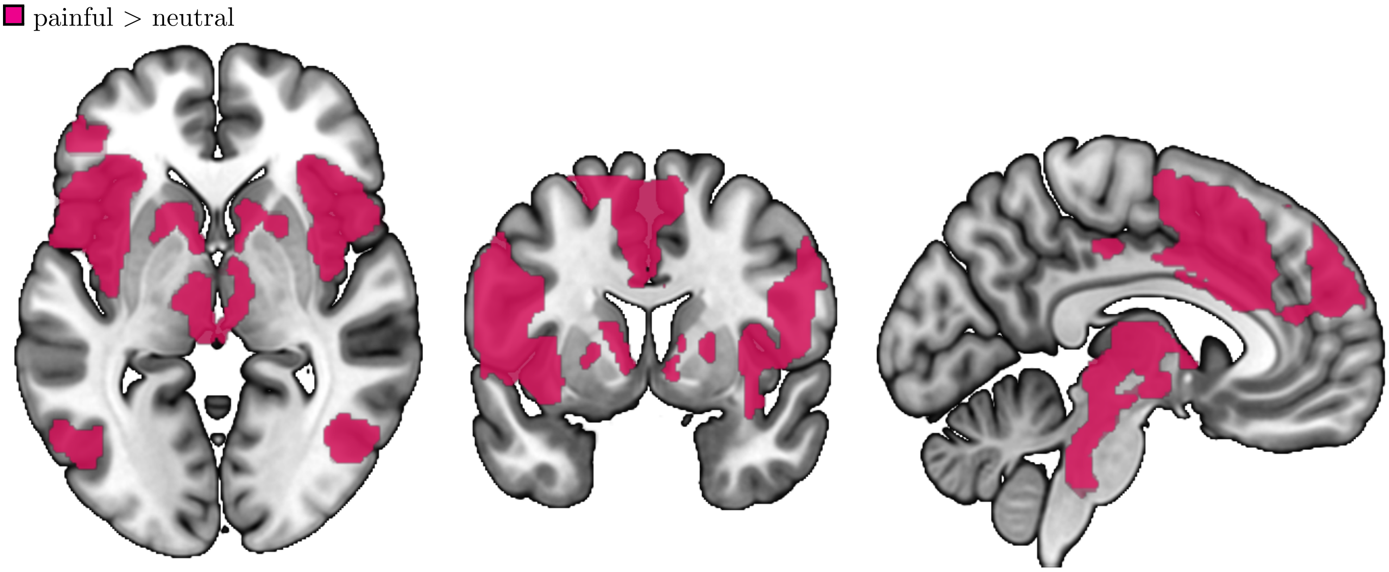

Figure S1: Binary visualisation of the significant clusters for the brain response to painful over neutral scenarios of mothers and non-mothers to both child and adult stimuli based on the analysis described in table S2.

Table S1: Results of the Bayesian mixed ANOVA of the painfulness ratings in the scanner.

Models	P(M)	P(M|data)	BF_M	BF_{10}	error %
Null model (incl. subject)	0.053	1.741e-92	3.134e-91	1.000	
pain	0.053	0.575	24.305	3.300e+91	0.838
pain + protagonist	0.053	0.150	3.175	8.613e+90	1.301
pain + mother	0.053	0.137	2.859	7.872e+90	1.410
pain + protagonist + pain * protagonist	0.053	0.038	0.707	2.171e+90	3.928
pain + protagonist + mother	0.053	0.036	0.663	2.042e+90	2.040
pain + mother + pain * mother	0.053	0.029	0.545	1.688e+90	1.672
pain + protagonist + mother + protagonist * mother	0.053	0.010	0.189	5.983e+89	5.776
pain + protagonist + mother + pain * protagonist	0.053	0.010	0.173	5.473e+89	9.783
pain + protagonist + mother + pain * mother	0.053	0.008	0.142	4.486e+89	2.152
pain + protagonist + mother + pain * protagonist + pain * mother	0.053	0.003	0.047	1.480e+89	17.224
pain + protagonist + mother + pain * protagonist + protagonist * mother	0.053	0.002	0.044	1.396e+89	4.476
pain + protagonist + mother + pain * mother + protagonist * mother	0.053	0.002	0.040	1.261e+89	2.954
pain + protagonist + mother + pain * protagonist + pain * mother + protagonist * mother	0.053	5.286e-4	0.010	3.036e+88	4.085
pain + protagonist + mother + pain * protagonist + pain * mother + protagonist * mother + pain * protagonist * mother	0.053	2.484e-4	0.004	1.427e+88	4.307
mother	0.053	3.074e-93	5.534e-92	0.177	0.836
protagonist	0.053	2.845e-93	5.121e-92	0.163	1.363
protagonist + mother	0.053	4.952e-94	8.914e-93	0.028	1.389
protagonist + mother + protagonist * mother	0.053	1.815e-94	3.267e-93	0.010	41.187
**Effects**	**P(incl)**	**P(excl)**	**P(incl|data)**	**BF_{incl}**	**$CI_{90\%}$ of η_p^2**
pain	0.737	0.263	1.000	5.361e+14	0.85-0.90
protagonist	0.737	0.263	0.259	0.125	0.00-0.04
mother	0.737	0.263	0.238	0.111	0.00-0.03
pain * protagonist	0.316	0.684	0.053	0.122	0.00-0.03
pain * mother	0.316	0.684	0.043	0.097	0.00-0.03
protagonist * mother	0.316	0.684	0.016	0.035	0.00-0.04
pain * protagonist * mother	0.053	0.947	2.484e-4	0.004	0.00-0.04

Table S2: Whole brain results of a one sample t-test pooling mothers and non-mothers based on differential first-level contrast images of "pain > neutral" pictures including both protagonists.

	BA	h	k_E	t-value	x	y	z
painful > neutral							
Supramarginal gyrus inc. anterior cingulate cortex	40	L	7515	11.54	-63	-23	38
	40			8.72	-67	-23	22
	1			8.01	-49	-41	60
Insula	45	L	4830	10.48	-43	18	4
	13			9.90	-37	-3	14
	13			9.69	-39	2	2
Postcentral gyrus	1	R	1258	9.68	62	-19	38
	40			6.26	56	-33	54
	40			5.11	66	-33	30
Cerebellum (Lob-6)		R	1139	8.32	36	-53	-31
				7.99	26	-69	-25
				5.79	20	-53	-25
Inferior frontal gyrus inc. insula	44	R	1948	8.17	50	8	16
	44			7.53	50	10	8
	13			7.34	40	-1	-1
Thalamus		L	1719	6.61	-7	-23	-3
				6.37	-11	-13	-1
				6.02	-5	-33	-27
Middle temporal gyrus	19	R	261	5.73	46	-69	-1
Precentral gyrus	6	R	120	5.13	38	-11	64
Cerebellum (Lob-6)		L	164	4.74	-31	-63	-25
				4.65	-35	-57	-29
				4.03	-19	-71	-23
Middle occipital gyrus	19	L	168	4.17	-45	-67	-1
	19			3.97	-55	-65	2

Cluster-corrected $p_{\mathrm{FWE}} < 0.05$; all results are grey matter masked; BA = Brodmann Area, L = left, R = right, M = medial, k_E = cluster size, coordinates are in MNI space and are the location of the peak voxel for each cluster.

Preprocessing: automatic fMRIPrep boilerplate text

Results included in this manuscript come from preprocessing performed using *fMRIPrep* 20.0.6 [2, 3], which is based on *Nipype* 1.4.2 [4, 5].

Anatomical data preprocessing The T1-weighted (T1w) image was corrected for intensity non-uniformity (INU) with `N4BiasFieldCorrection` [6], distributed with ANTs 2.2.0 [7], and used as T1w-reference throughout the workflow. The T1w-reference was then skull-stripped with a *Nipype* implementation of the `antsBrainExtraction.sh` workflow (from ANTs), using OASIS30ANTs as target template. Brain tissue segmentation of cerebrospinal fluid (CSF), white-matter (WM) and gray-matter (GM) was performed on the brain-extracted T1w using `fast` [8]. Brain surfaces were reconstructed using `recon-all` [9], and the brain mask estimated previously was refined with a custom variation of the method to reconcile ANTs-derived and FreeSurfer-derived segmentations of the cortical gray-matter of Mindboggle [10]. Volume-based spatial normalization to one standard space (MNI152NLin2009cAsym) was performed through nonlinear registration with `antsRegistration` (ANTs 2.2.0), using brain-extracted versions of both T1w reference and the T1w template. The following template was selected for spatial normalization: *ICBM 152 Nonlinear Asymmetrical template version 2009c* [[11], RRID: SCR_008796; TemplateFlow ID: MNI152NLin2009cAsym],

Functional data preprocessing For each of the 6 BOLD runs found per subject (across all tasks and sessions), the following preprocessing was performed. First, a reference volume and its skull-stripped version were generated using a custom methodology of *fMRIPrep*. A B0-nonuniformity map (or *fieldmap*) was estimated based on a phase-difference map calculated with a dual-echo GRE (gradient-recall echo) sequence, processed with a custom workflow of *SDCFlows* inspired by the `epidewarp.fsl` script and further improvements in HCP Pipelines [12]. The *fieldmap* was then co-registered to the target EPI (echo-planar imaging) reference run and converted to a displacements field map (amenable to registration tools such as ANTs) with FSL's `fugue` and other *SDCflows* tools. Based on the estimated susceptibility distortion, a corrected EPI (echo-planar imaging) reference was calculated for a more accurate co-registration with the anatomical reference. The BOLD reference was then co-registered to the T1w reference using `bbregister` (FreeSurfer) which implements boundary-based registration [13]. Co-registration was configured with six degrees of freedom. Head-motion parameters with respect to the BOLD reference (transformation matrices, and six corresponding rotation and translation parameters) are estimated before any spatiotemporal filtering using `mcflirt` [14]. BOLD runs were slice-time corrected using `3dTshift` from AFNI 20160207 [15]. The BOLD time-series (including slice-timing correction when applied) were resampled onto their original, native space by applying a single, composite transform to correct for head-motion and susceptibility distortions. These resampled BOLD time-series will be referred to as *preprocessed BOLD in original space* or just *preprocessed BOLD*. The BOLD time-series were resampled into standard space, generating a *preprocessed BOLD run in MNI152NLin2009cAsym space*. First, a reference volume and its skull-stripped version were generated using a custom methodology of *fMRIPrep*. Several confounding time-series were calculated based on the *preprocessed BOLD*: framewise displacement (FD), DVARS and three region-wise global signals. FD and DVARS are calculated for each functional run, both using their implementations in *Nipype* [16]. The three global signals are extracted within the CSF, the WM and the whole-brain masks. Additionally, a set of physiological regressors were extracted to allow for component-based noise correction [17]. Principal components are estimated after high-pass filtering the *preprocessed BOLD* time-series (using a discrete cosine filter with 128s cut-off) for the two *CompCor* variants: temporal (tCompCor)

and anatomical (aCompCor). tCompCor components are then calculated from the top 5% variable voxels within a mask covering the subcortical regions. This subcortical mask is obtained by heavily eroding the brain mask, which ensures it does not include cortical GM regions. For aCompCor, components are calculated within the intersection of the aforementioned mask and the union of CSF and WM masks calculated in T1w space, after their projection to the native space of each functional run (using the inverse BOLD-to-T1w transformation). Components are also calculated separately within the WM and CSF masks. For each CompCor decomposition, the k components with the largest singular values are retained, such that the retained components' time series are sufficient to explain 50 percent of variance across the nuisance mask (CSF, WM, combined or temporal). The remaining components are dropped from consideration. The head-motion estimates calculated in the correction step were also placed within the corresponding confounds file. The confound time series derived from head motion estimates and global signals were expanded with the inclusion of temporal derivatives and quadratic terms for each [18]. Frames that exceeded a threshold of 0.5 mm FD or 1.5 standardised DVARS were annotated as motion outliers. All resamplings can be performed with *a single interpolation step* by composing all the pertinent transformations (i.e. head-motion transform matrices, susceptibility distortion correction when available and co-registrations to anatomical and output spaces). Gridded (volumetric) resamplings were performed using `antsApplyTransforms` (ANTs), configured with Lanczos interpolation to minimize the smoothing effects of other kernels [19]. Non-gridded (surface) resamplings were performed using `mri_vol2surf` (FreeSurfer).

Many internal operations of *fMRIPrep* use *Nilearn* 0.6.2 [20], mostly within the functional processing workflow. For more details of the pipeline, see the section corresponding to workflows in *fMRIPrep*'s documentation.

Copyright waiver The above boilerplate text was automatically generated by fMRIPrep with the express intention that users should copy and paste this text into their manuscripts *unchanged*. It is released under the CC0 license.

References

[1] JASP Team. JASP (Version 0.14)[Computer software] (2020). URL https://jasp-stats.org/.

[2] Esteban, O. *et al.* fMRIPrep: a robust preprocessing pipeline for functional MRI. *Nature Methods* (2018).

[3] Esteban, O. *et al.* fmriprep. *Software* (2018).

[4] Gorgolewski, K. *et al.* Nipype: a flexible, lightweight and extensible neuroimaging data processing framework in python. *Frontiers in Neuroinformatics* **5**, 13 (2011).

[5] Gorgolewski, K. J. *et al.* Nipype. *Software* (2018).

[6] Tustison, N. J. *et al.* N4itk: Improved n3 bias correction. *IEEE Transactions on Medical Imaging* **29**, 1310–1320 (2010).

[7] Avants, B., Epstein, C., Grossman, M. & Gee, J. Symmetric diffeomorphic image registration with cross-correlation: Evaluating automated labeling of elderly and neurodegenerative brain. *Medical Image Analysis* **12**, 26–41 (2008). URL http://www.sciencedirect.com/science/article/pii/S1361841507000606.

[8] Zhang, Y., Brady, M. & Smith, S. Segmentation of brain MR images through a hidden markov random field model and the expectation-maximization algorithm. *IEEE Transactions on Medical Imaging* **20**, 45–57 (2001).

[9] Dale, A. M., Fischl, B. & Sereno, M. I. Cortical surface-based analysis: I. segmentation and surface reconstruction. *NeuroImage* **9**, 179–194 (1999). URL http://www.sciencedirect.com/science/article/pii/S1053811998903950.

[10] Klein, A. *et al.* Mindboggling morphometry of human brains. *PLOS Computational Biology* **13**, e1005350 (2017). URL http://journals.plos.org/ploscompbiol/article?id=10.1371/journal.pcbi.1005350.

[11] Fonov, V., Evans, A., McKinstry, R., Almli, C. & Collins, D. Unbiased nonlinear average age-appropriate brain templates from birth to adulthood. *NeuroImage* **47, Supplement 1**, S102 (2009).

[12] Glasser, M. F. *et al.* The minimal preprocessing pipelines for the human connectome project. *NeuroImage* **80**, 105–124 (2013). URL http://www.sciencedirect.com/science/article/pii/S1053811913005053.

[13] Greve, D. N. & Fischl, B. Accurate and robust brain image alignment using boundary-based registration. *NeuroImage* **48**, 63–72 (2009).

[14] Jenkinson, M., Bannister, P., Brady, M. & Smith, S. Improved optimization for the robust and accurate linear registration and motion correction of brain images. *NeuroImage* **17**, 825–841 (2002). URL http://www.sciencedirect.com/science/article/pii/S1053811902911328.

[15] Cox, R. W. & Hyde, J. S. Software tools for analysis and visualization of fmri data. *NMR in Biomedicine* **10**, 171–178 (1997).

[16] Power, J. D. *et al.* Methods to detect, characterize, and remove motion artifact in resting state fmri. *NeuroImage* **84**, 320–341 (2014). URL http://www.sciencedirect.com/science/article/pii/S1053811913009117.

[17] Behzadi, Y., Restom, K., Liau, J. & Liu, T. T. A component based noise correction method (CompCor) for BOLD and perfusion based fmri. *NeuroImage* **37**, 90–101 (2007). URL http://www.sciencedirect.com/science/article/pii/S1053811907003837.

[18] Satterthwaite, T. D. *et al.* An improved framework for confound regression and filtering for control of motion artifact in the preprocessing of resting-state functional connectivity data. *NeuroImage* **64**, 240–256 (2013). URL http://linkinghub.elsevier.com/retrieve/pii/S1053811912008609.

[19] Lanczos, C. Evaluation of noisy data. *Journal of the Society for Industrial and Applied Mathematics Series B Numerical Analysis* **1**, 76–85 (1964). URL http://epubs.siam.org/doi/10.1137/0701007.

[20] Abraham, A. *et al.* Machine learning for neuroimaging with scikit-learn. *Frontiers in Neuroinformatics* **8** (2014). URL https://www.frontiersin.org/articles/10.3389/fninf.2014.00014/full.
